# Nurses' Emotional Intelligence Impact on the Quality of Hospital Services

**DOI:** 10.5812/ircmj.926

**Published:** 2012-12-06

**Authors:** Mohammad Ranjbar Ezzatabadi, Mohammad Amin Bahrami, Farzaneh Hadizadeh, Masoomeh Arab, Soheyla Nasiri, Mohammadreza Amiresmaili, Gholamreza Ahmadi Tehrani

**Affiliations:** 1 Shahid Sadoughi University of Medical Sciences, Yazd, IR Iran; 2 Medical Informatics Research Center, Kerman University of Medical Sciences, Kerman, IR Iran

**Keywords:** Emotional Intelligence, Job Satisfaction, Communication

## Abstract

**Background:**

Emotional intelligence is the potential to feel, use, communicate, recognize, remember, describe, identify, learn from, manage, understand and explain emotions. Service quality also can be defined as the post-consumption assessment of the services by consumers that are determined by many variables.

**Objectives:**

This study was aimed to determine the nurses’ emotional intelligence impact on the delivered services quality.

**Materials and Methods:**

This descriptive - applied study was carried out through a cross-sectional method in 2010. The research had 2 populations comprising of patients admitted to three academic hospitals of Yazd and the hospital nurses. Sample size was calculated by sample size formula for unlimited (patients) and limited (nursing staff) populations and obtained with stratified- random method. The data was collected by 4 valid questionnaires.

**Results:**

The results of study indicated that nurses' emotional intelligence has a direct effect on the hospital services quality. The study also revealed that nurse's job satisfaction and communication skills have an intermediate role in the emotional intelligence and service quality relation.

**Conclusions:**

This paper reports a new determinant of hospital services quality.

## 1. Background

### 1.1. Emotional Intelligence

While cognitive intelligence is primarily associated with memory and problem solving capacity, the founders of the modern concept of intelligence recognized the possibility of non-cognitive forms (1, 2). More recently, Salovey and Mayer, utilizing scientific methods, operationalized a concept which they referred to as emotional intelligence (EI) (3). EI includes the ability to monitor one’s own and others’ feelings and emotions, to discriminate among them and to use this information to guide one’s thinking and actions, also the capacity to perceive emotions, assimilate emotion-related feelings, understand the information of these emotions and manage them (4).It is interesting to note that there is a growing literature relating to emotional aspects of organizational life (5). Although, few management researchers have embraced the concept but the notion of emotional intelligence with its vast applicability to many workplace issues including performance, job satisfaction, absenteeism, organizational commitment and leadership issues has considerably appeal with practitioners (6). Researches have been indicated a correlation between EI and top performers and performance climates in the workplace. According to such researches, EI is supported as a vital element in excellent job performance profiles, in employee behavior and organizational practices leading to an outstanding climate for service delivery and in employee concern for quality and ability to deal with workplace conflict. So a considerable amount of literature advocates for EI as a key ingredient on which human resource professionals and organizations must be focused (7).

### 1.2. Servqual

As it is well known, the quality is accepted as being an important factor that determines the demand of goods and services as well as a main indicator that affects the competitive advantage of firms. The term of quality in the service sector seems to be different from the term in the goods market. Since the production in the service sector is generally an abstract term, evaluating service quality becomes more difficult than evaluating the quality of goods. Therefore, service quality measurements are, in general, made by means of using consumers’ (patients) perception about the quality of the services (8). During previous decades, service quality (SERVQUAL) has been became a significant issue in management studies and many tools have been developed to appraise it. Such tools are different in terms of definition, content and type of appraisal. However SERVQUAL measurement that was designed by the marketing team of Parasuraman, Zeithmal and Berry (1988) is the most common tool for measurement of service quality. It examines SERVQUAL through comparing customers' expectations and perceptions in various dimensions (9).According to this scale, if the performance exceeds expectations the consumer will attain more satisfaction (8).

There are several factors that affect service quality such as high level manager's support, proper investment, personal efforts, etc. Among other things, EI of service providers seems to be one of the most important determinants of SERVQUAL. Service providers' emotional intelligence can affect service quality, either directly or through other mediator variables such as individual's communication skills; his/her job satisfaction, conflict management strategies and organizational commitment of providers (10). Given the mentioned history, in this study, we have tried to investigate the nurses’ emotional intelligence impact on the delivered services quality in teaching hospitals. For that we hypothesized that nurses’ emotional intelligence status has direct relationship with the delivered services quality. Also, we hypothesized that nurses’ communication skills and their job satisfaction have the intermediate role in the relationship of nurses’ emotional intelligence and services quality.

## 2. Objective

This study was aimed to determine the nurses’ emotional intelligence impact on the delivered services quality.

## 3. Materials and Methods

**Table 1:** Background Characteristics of Research Sample

	N0. (%)
**Patients**	193
**Gender**	
Male	110 (57)
Female	83 (43)
**Marital Status**	
Single	57 (30)
Married	136 (70)
**Age**	
<30	112 (58)
30–40	29 (15)
>40	52 (27)
**Nurses**	243
**Gender**	
Male	35 (14)
Female	208 (86)
**Marital Status**	
Single	62 (26)
Married	181(74)
**Age**	
< 30	126 (52)
30–40	89 (37)
> 40	28 (11)

This descriptive - applied study was carried out through a cross-sectional method in 2010. It had two populations including nurses and in-patients of three academic hospitals of Yazd, Iran. We used stratified-random sampling method. The sample Size of limited population (nursing staff) and unlimited population (patients) were calculated vian=(NT^z^pq)/(T^z^ pq+Nd^z^ ) and n=(T^z^ pq)/(d^z^) respectively, with the confidence level of %95 and the confidence interval of /1. In total, 193 patients and 243 nursing staff participated in the study. Background characteristics of samples are presented in Table 1.The required data of study was collected using following instruments.

### 3.1. Servqual

For this part of study, the 22 items SERVQUAL questionnaire developed by Parasuraman et al. was used (11). Some modifications and adaptations were made to select questions to make them more relevant to the study. This questionnaire which consisted of 22 questions in five dimensions (tangibility, reliability, responsiveness, assurance and empathy), were given to in- patients of hospitals. The questionnaire contained an "expectations" section with 22 statements and a "perceptions" section consisting of a set of matching statements. The statements in both the expectations and perceptions sections were grouped into the above five dimensions each with a range of applicable statements. The 5 points Likert scale was used for the scoring system with 1 representing "strongly disagree" and 5 representing "strongly agree".

### 3.2. Emotional Intelligence

Emotional intelligence of nursing staff was surveyed with Cyberia Shrink 33 items questionnaire (12). This instrument evaluates emotional intelligence in five dimensions including self-awareness, self-control, self-motivation, empathy and social skills. A 5points Likert scale was used for the scoring system. So, each nurse could obtain a total score between 33 and 165 that was divided to 33 for obtaining his/her emotional intelligence final score. In this questionnaire, items were written in both directions, so 11 questions must be reverse scored. After completing the questionnaires by the nursing staff, their emotional intelligence was calculated by using mean and standard deviation measures.

### 3.3. Job Satisfaction

The job satisfaction of nursing staff was measured by Job Satisfaction Survey (13). The Job Satisfaction Survey (JSS) is a 36 item, 9 facet scales to assess employee attitudes about the job and aspects of the job. Each facet is assessed with 4 items, and a total score is computed from all items. A summated rating scale format is used, with 5 choices per item ranging from "disagree very much" to "agree very much". Items are written in both directions, so about half must be reverse scored. The 9 facets are Pay, Promotion, Supervision, Fringe Benefits, Contingent Rewards (performance based rewards), Operating Procedures (required rules and procedures), Coworkers, Nature of Work and Communication. The total job satisfaction of nursing staff was calculated by using mean and standard deviation measures.

### 3.4. Communication Skills

Communication skills in nursing staff have some unique aspects. It is difficult to design instruments for objective evaluation of this competency. With extensive literature review, we developed a questionnaire that focused communication skills that should be addressed in nursing practices. The questionnaire consisted of 17 items that covered the important components of the communication skills including clarity of communication; rapport with patients, patients’ families, physician and non-physician personnel; listening skills; management skills and respect for others. Each item was measured on a 9points Likert scale (1, unsatisfactory performance to 9, outstanding performance). We used 360-degree approach for the nursing staff’s evaluation. We created a survey prior to implement the evaluation. This survey allowed us to retrieve input and opinions from nursing staff concerning this new format for competency assessment. Before initiating the study, all nursing staff were informed of the 360-degree evaluation and were asked to participate in this evaluation process. The rater categories included nurses, other health professionals, secretarial staffs, patients and their families. The raters were oriented and instructed in the use of the evaluation form by one of the authors before implementation. 

Rating forms were distributed to raters. Completed forms were analyzed and the communication skills competency of nurses evaluated by calculating mean of all scores that they obtained. The validity of questionnaires was gained using experts' viewpoints (content validity) and factor analysis test (structure validity). Their reliability also was obtained through calculating Chronbach's Alpha coefficient. The reliability coefficients of the questionnaires are summarized in Table 2.

**Table 2:** Reliability Coefficients of Questionnaires

roup, Questionnaire	Hospital	Chronbach's Alpha coefficient
**Patients**		
Servqual	Shahid Sadoughi	0.94
	Shahid Rahnemoon	0.94
	Afshar	0.94
	Total	0.95
**Nursing Staff**		
Emotional Intelligence	Shahid Sadoughi	0.79
	Shahid Rahnemoon	0.83
	Afshar	0.84
	Total	0.81
Communication Skills	Shahid Sadoughi	0.84
	Shahid Rahnemoon	0.86
	Afshar	0.77
	Total	0.83
Job Satisfaction	Shahid Sadoughi	0.82
	Shahid Rahnemoon	0.83
	Afshar	0.83
	Total	0.84

**Figure 1:**
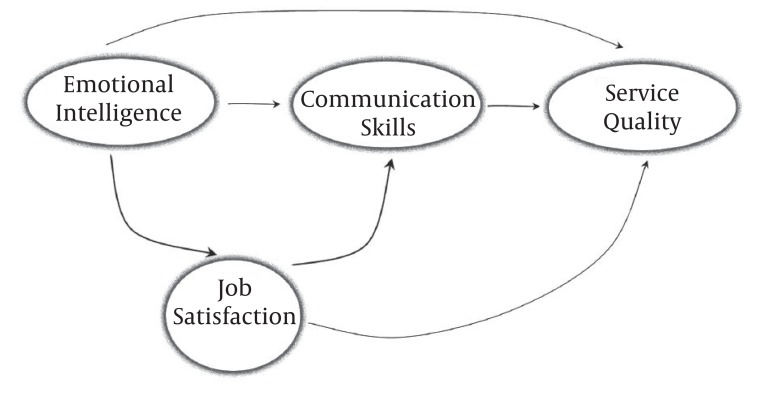
Theoretical Model of Research

We used SPSS 11.5 and LISREL_8_ software in data analyzing. In SERVQUAL analysis we used Kolmogorov-Smirnov test for testing the normal distribution of observations and paired sample T-test for testing the significance of SERVQUAL gap (differences between patients’ expectations and perceptions from services quality). Also, descriptive statistics including mean and standard deviation were used for analyzing obtained data of nursing staffs’ emotional intelligence, communication skills and job satisfaction. Finally, the hypotheses of research were tested by using T-value amount. These hypotheses can be observed in the designed theoretical model of research as Figure 1.

## 4. Results

In order to testing the research hypotheses and exacting final model of research, we surveyed the quality of offered services of hospitals. Also, we calculated the emotional intelligence, communication skills and job satisfaction of employed nursing staff. Table 3 shows the expectations – perceptions gap of patients in each hospital.

**Table 3:** The Results of Servqual Gap Analysis

Hospital	Mean	Standard Error Mean	Standard Deviation	Confidence Level %95		t	Df.	Sig.
				Low	High			
**Shahid Sadoughi (E_1_-P_1_)**	1.13	0.04	0.22	1.04	1.23	24.02	21	(P = 0.00)
**Shahid Rahnemoon (E_2_-P_2_)**	1.71	0.06	0.29	1.58	1.84	27.33	21	(P = 0.00)
**Afshar (E_3_-P_3_)**	1.27	0.05	0.05	1.16	1.38	24.50	21	(P = 0.00)

Table 3 indicates the existence of SERVQUAL gap in all three hospitals. Also, Table 4 shows the mean and standard deviation of emotional intelligence, job satisfaction and communication skills of nursing staff in all hospitals.

**Table 4:** The Emotional Intelligence, Job Satisfaction and Communication Skills of Nursing Staff

	Emotional intelligence, Mean ± SD	Job Satisfaction, Mean ± SD	Communication Skills, Mean ± SD
**Shahid Sadoughi**	3.88 ± 0.58	3.17 ± 1.73	8.13 ± 1.29
**Shahid Rahnemoon**	3.41 ± 0.41	2.74 ± 0.98	6.62 ± 1.54
**Afshar**	4.30 ± 0.50	3.26 ± 1.35	8.97 ± 1.84

**Figure 2:**
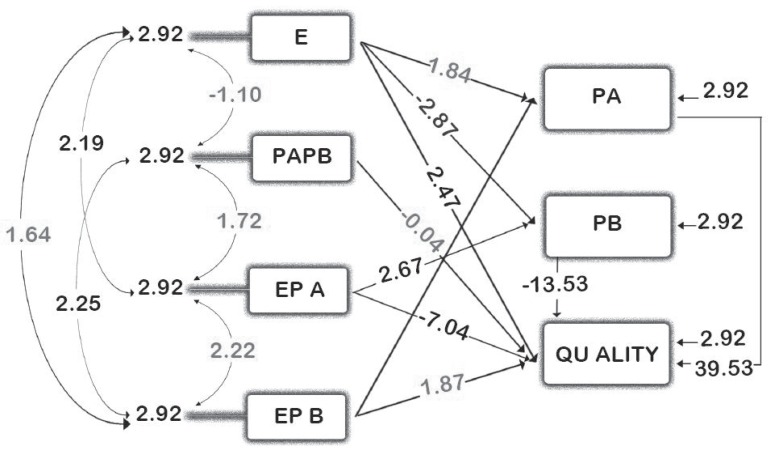
T-Value Amounts in Variables Relationships

**Figure 3:**
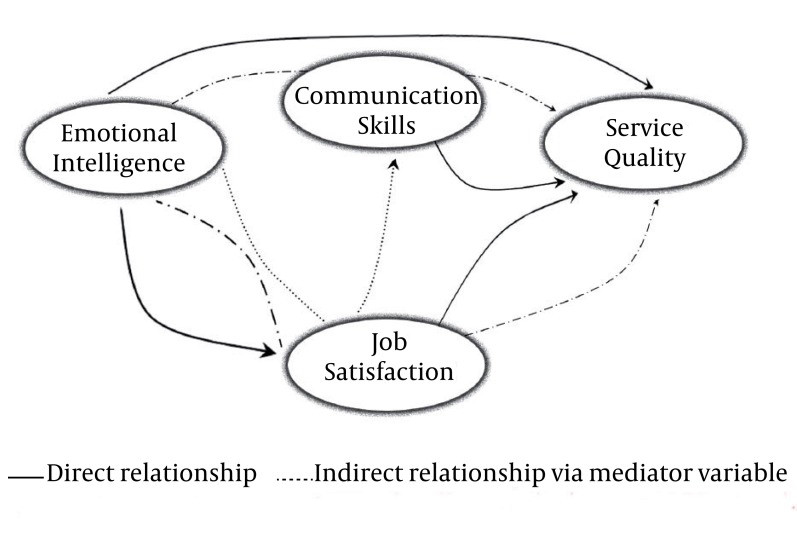
Approved Model of Nursing Staff EI Effect on the Hospital Servqual

After surveying the research variables (SERVQUAL, Emotional Intelligence, Job Satisfaction and Communication Skills) we tested the research hypotheses trough T-value amount (Figure 2) and exacted the approved model which presented in Figure 3.

## 5. Discussion

There are a set of competencies that have been shown to cause or predict outstanding performance. Regardless of author or study, they tend to include abilities from three clusters: cognitive, emotional and social intelligence competencies (13). These competencies predict effectiveness in professional, management and leadership roles in many sectors of society (14). Emotional intelligence is very important in the workplace. Ciarrochi et al. stress that being able to understand, perceive and express emotions in an appropriate way can determine whether an individual is successful or not as an employee in a career (15). Therefore, EI is becoming one of the most important individual competencies for organizations and has been theoretically related to organizational performance and to individual variables like job satisfaction(16). This study was aimed to investigate the impact of nursing staffs’ emotional intelligence on the delivered services quality. Based on the expanding literature review, we hypothesized that some individual variables including communication skills and job satisfaction act as a mediator variable in relation of EI and SERVQUAL. The results indicated that nursing staffs’ emotional intelligence has a direct effect on the offered services quality. This means that emotionally intelligent nursing staffs deliver more qualified services and perform beyond the patients’ expectations from service quality that, in turn leads to patients’ loyalty, guarantees their purchases and affects consumer behavior positively.

Some another researchers examined such relationship between EI and quality of services. Among their, Rao, Kiely, Fer, Deadrick and Bruce and Bardzil and Slaski have shown that EI has positive relationship with SERVQUAL and play a significant role to improve general performance, productivity, preserving and attracting customers. They also found that EI may effect on creation and maintenance of quality culture, service-related skills learning, work success and patients' satisfaction (17-22). Also, we hypothesized that job satisfaction is a mediator variable in relation of emotional intelligence and service quality. Job satisfaction is normally defined as an employee’s affective reactions to a job based on a comparison of desired outcomes and actual outcomes (22). There are important reasons why we should be concerned with job satisfaction. One of the most important is that job satisfaction can lead to employee behaviors that affect on organizational functioning and performance (23). The situational approach to job satisfaction considers that job satisfaction is primarily determined by the characteristics of the work. However, the dispositional approach understands affective dispositions to be the prime determinant of job satisfaction. Job satisfaction may be affected by emotion-related personality traits because it has been equated with a pleasurable emotional state (16).

The results of the study showed that emotional intelligence has a direct relation with job satisfaction. Also, job satisfaction has a mediator role in the emotional intelligence and service quality relation and affects service quality directly. These results can be expressed so that emotionally intelligent people seem to be satisfied with their jobs more than others and the more job satisfaction, in return, affects the quality of delivered services positively. Some studies such as Tram and O'hara and Gannon and Ranijnt also found that there is a positive correlation between EI and job satisfaction (24, 25). Which were proved in this study too? Finally, we hypothesized that emotional intelligence competency can affect the communication skills of nursing staff and the latest affects services quality. Some literatures have been proposed that the awareness of one’s own and others’ emotions enables individuals to establish sound interpersonal relationships with others (26). People with this ability, therefore, should be able to recognize and understand what their emotions are and know how to apply them in improving their relationships with others (27). Such improved relationships may in turn lead to improved job performance (28).

High levels of EI have also been shown to affect individual communication skills. In this case, individuals regard their own emotions and the emotions of others as a basis to determine which styles are appropriate in communicating with others (29). Yousefi, Shagholian, Nikoo Goftar, Vatankhah et al. Besharat and Lopez et al. have indicated that individuals' EI level may effect on their communication skills and conflict management strategies, directly (30-34). Despite this literature, our research didn’t show the same relationship. Based on the results, emotional intelligence level has no direct effect on the individual’s communication skills. In the same time, we indicated that communication skills have a mediator role in the relation of emotional intelligence and service quality. Also, job satisfaction has a mediator role in the relation of emotional intelligence and communication skills. Finally, communication skills affect the service quality directly. We can conclude that, although emotional intelligence don’t affect the communication skills of nursing staff but it can affect it through improving job satisfaction that eventually will lead to perform better in regarding to high quality services delivery.

The applicability of our study is strengthened with the notion that the emotional intelligence competency can be developed in people. Decades of research on the effects of psychotherapy, self-help programs, cognitive behavior therapy, training programs and education have shown that people can change their behavior, moods, and self-image (35). Therefore, emotional intelligence competency needs to be managed through a certain contextual, organizational or managerial situation (29). In order to suggest some implications for human resource management, we might conclude that emotionally intelligent nursing staffs are more likely to deliver high quality services that aimed in many managerial initiatives in the health sector recently.
